# Supplementary material for: Phase separation of BuGZ regulates gut regeneration and aging through interaction with m6A regulators
Source: Nat Commun. 2023 Oct 23;14:6700. doi: 10.1038/s41467-023-42474-1 (PMC10593810; doi:10.1038/s41467-023-42474-1)
Supplement: Supplementary file 1 — Supplementary Information [file 41467_2023_42474_MOESM1_ESM.pdf]

Supplementary Information for

Phase separation of BuGZ regulates gut regeneration and aging through interaction with m^6A regulators

Qiaoqiao Zhang, Kai Deng, Mengyou Liu, Shengye Yang, Wei Xu, Tong Feng, Minwen Jie, Zhiming Liu, Xiao Sheng, and Haiyang Chen*, and Hao Jiang*

This PDF file includes: Supplementary Figures 1 to 10

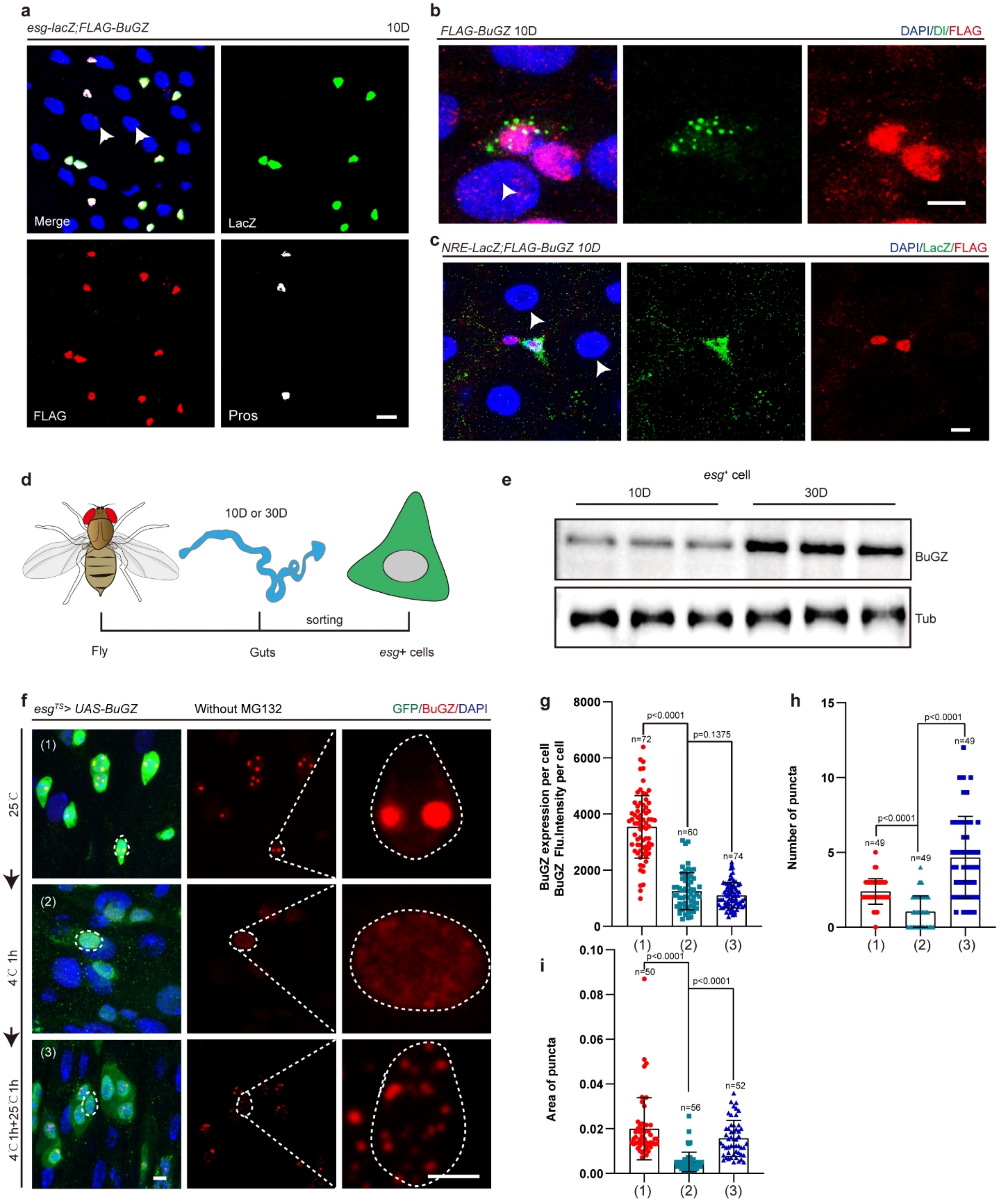

Fig. 1. BuGZ locates in fly gut diploid cells and its level increases in old *esg*+ cells. a, b, c BuGZ locates in diploid cells (*esg*-LacZ labelled stem cell and progenitor cells (**a**), Dl (Delta) labelled ISCs (**b**), NRE-LacZ labelled EB cells (**c**), Pros labels EE cells (**a**), but not in polyploid

ECs in young (10D) midguts. White arrows indicate ECs. DAPI labels the nucleus. **d** Model of sorting *esg*+ cells in young (10D) and old (30D) midguts. **e** Western blotting shows elevated levels of dBuGZ protein in old (30D) sorted esg^+ cells compared to young (10D) esg^+ cells. **f, g, h, i** The change of BuGZ puncta at low temperature without adding MG132. Immunofluorescence intensity analyses showed that BuGZ protein level decreased at low temperature without MG132 treatment, whereas BuGZ puncta still diffused when the midguts were transferred from 25℃ to 4℃. Moreover, the diffused BuGZ protein concentrated again to form more but smaller puncta when these low temperature-cultured midguts were transferred back to 25℃. Intensity (**g,** from left to right are 72, 60, 74 cells), number (**h,** from left to right are 49, 49, 49 cells), area (**i,** from left to right are 50, 56, 52 cells) of BuGZ puncta. GFP indicated esg^+ cells. DAPI stained nuclei (blue). Scale bars represent 5 μm (**b**, **c**, **f**), 10μm (**a**). Bars are mean ± SD. *P* values were calculated by two-tailed, unpaired Student's *t* test. Source data are provided as a Source Data file.

.

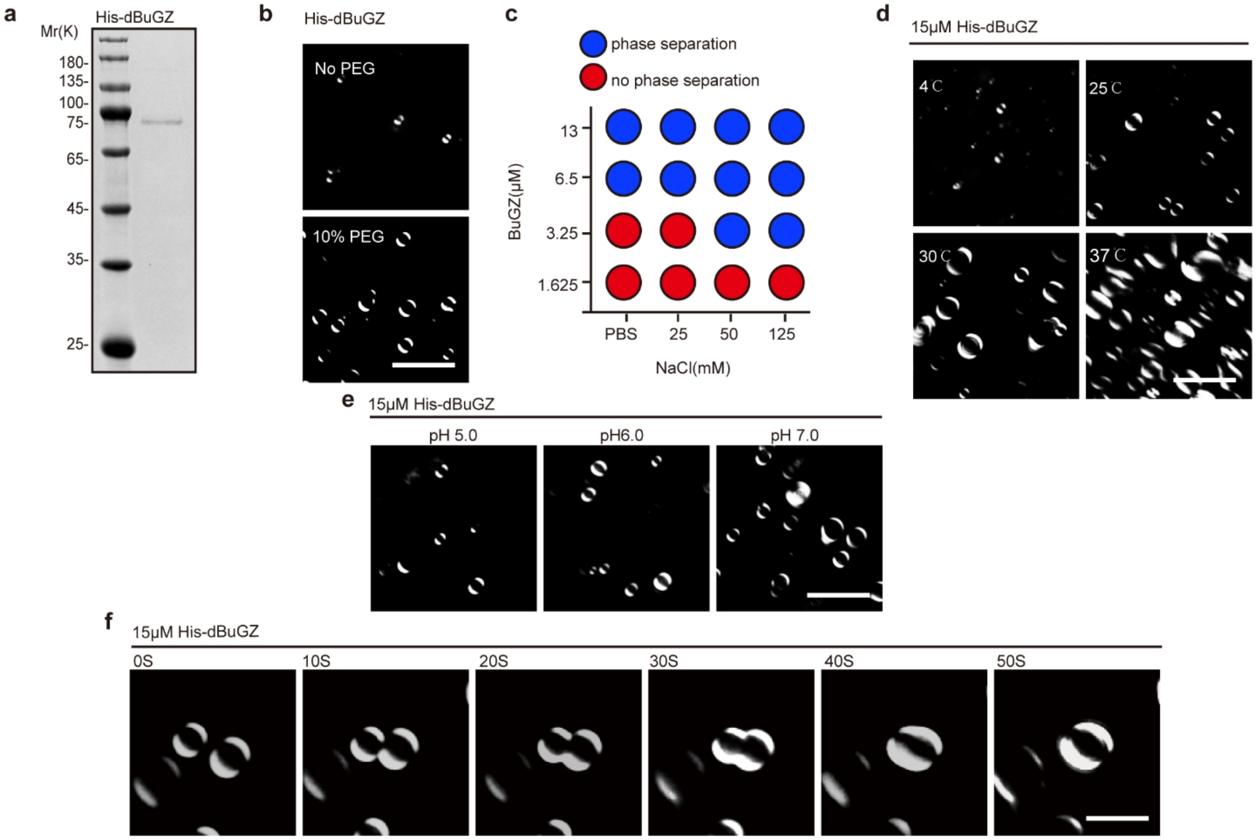

Fig. 2. BuGZ undergoes phase separation *in vitro*. a SDS-PAGE shows purified His-tagged *Drosophila*-BuGZ protein from *E.coli*. **b** The droplets of dBuGZ were bigger with 10% PEG-6K. **c** Concentration- and salt hydronium concentration-dependent phase separation of His-dBuGZ. **d** Temperature-dependent phase separation of His-dBuGZ **e** pH conditions play a vital role in droplets of His-dBuGZ *in vitro*. **f** Fusion of droplets over time indicates the liquid-like property of His-dBuGZ droplets. Scale bars represent 5 μm (**f**), 10μm (**b**, **d**, **e**). Source data are provided as a Source Data file.

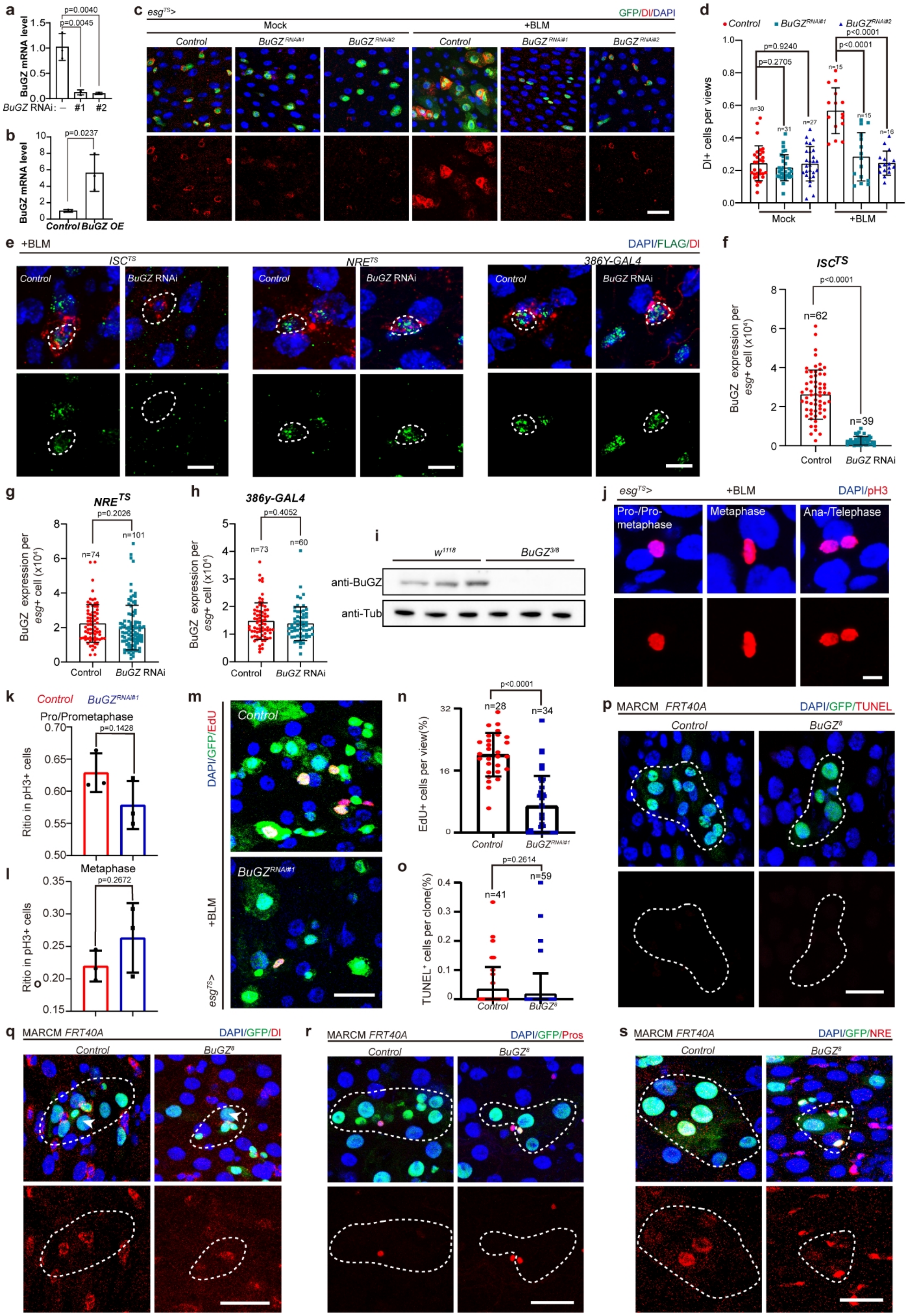

a
p=0.0040
p=0.0045
BuGZ mRNA level
BuGZ RNAi: — #1 #2
b
p=0.0237
BuGZ mRNA level
Control BuGZ OE
c
esg^TS>
GFP/Dl/DAPI
Mock
+BLM
Control
BuGZ RNAi#1
BuGZ RNAi#2
d
Control BuGZ RNAi#1 BuGZ RNAi#2
p=0.9240
p=0.2705
p<0.0001
p<0.0001
Dl+ cells per views
n=30 n=31 n=27 n=15 n=15 n=16
Mock
+BLM
e
+BLM
DAPI/FLAG/Dl
ISC^TS
NRE^TS
386Y-GAL4
Control
BuGZ RNAi
f
ISC^TS
p<0.0001
n=62
n=39
BuGZ expression per esg+ cell (x10^4)
Control BuGZ RNAi
g
NRE^TS
p=0.2026
n=74 n=101
BuGZ expression per esg+ cell (x10^4)
Control BuGZ RNAi
h
386y-GAL4
p=0.4052
n=73 n=60
BuGZ expression per esg+ cell (x10^4)
Control BuGZ RNAi
i
w^1118
BuGZ^3/8
anti-BuGZ
anti-Tub
j
esg^TS>
+BLM
DAPI/pH3
Pro-/Pro-metaphase
Metaphase
Ana-/Telephase
k
Control BuGZ RNAi#1
Pro/Prometaphase
p=0.1428
Ritio in pH3+ cells
l
Metaphase
p=0.2672
Ritio in pH3+ cells
m
DAPI/GFP/EdU
Control
BuGZ RNAi#1
+BLM
esg^TS>
n
p<0.0001
n=28 n=34
EdU+ cells per view(%)
Control BuGZ RNAi#1
o
p=0.2614
n=41 n=59
TUNEL+ cells per clone(%)
Control BuGZ^8
p
MARCM FRT40A
DAPI/GFP/TUNEL
Control
BuGZ^8
q
MARCM FRT40A
DAPI/GFP/Dl
Control
BuGZ^8
r
MARCM FRT40A
DAPI/GFP/Pros
Control
BuGZ^8
s
MARCM FRT40A
DAPI/GFP/NRE
Control
BuGZ^8

Fig. 3. BuGZ is indispensable for ISC proliferation during the S phase and might not be important for ISC differentiation. **a, b** Validation data for the expression changes of *BuGZ* RNAi or *BuGZ* overexpression. **c, d** BuGZ depletion in *esg*+ cells affected the number of Dl+ cells in injured young midguts, but not control ones. GFP indicated esg^{+} cells. **e, f, g, h** BuGZ depletions in ISCs diminished the BuGZ level in *esg*+ cells, whereas *BuGZ* RNAi in EEs or EBs did not affects BuGZ expression in *esg*+ cells. Dl (Delta) indicates ISCs. **i** Validation data of *BuGZ* knockout strains. **j, k, l** BuGZ regulates the proliferation of intestinal stem cells not by controlling the process of mitosis. Representative images indicate the pattern of different periods of $pH3^{+}$ and DAPI cells during mitosis (**j**). Ratio of prophase/prometaphase and metaphase period of $pH3^{+}$ cells to total $pH3^{+}$ cells in control (*UAS-LacZ*) and BuGZ-depleted esg^{+} cells (**k, l**). Error bars show the SD of three independent experiments. **m, n** BuGZ was not required for asymmetric division of ISCs but was essential for regulating the proliferation rate during the S phase. EdU immunostaining of midguts is shown in control (*UAS-LacZ*) and BuGZ-depleted esg^{+} cells after BLM treatment (**m**). Ratio of EdU^{+} cells to esg^{+} cells (**n**). (*Control*: n=28 views; $BuGZ^{RNAi}$: n=34 views). GFP indicates *esg*+ cells (green). **o, p** $TUNEL^{+}$ signals are shown in control and *BuGZ-null* MARCM clones (**p**). Ratio of $TUNEL^{+}$ cells to clone cells in control (*FRT40A*) and *BuGZ-null* MARCM clones (**o,** *Control*: n=41 clones; $BuGZ^{8}$: n=59 clones). GFP indicates ISCs (green). **q, r, s** BuGZ may not be important for ISC differentiation. Image of control (*FRT40A*) and *BuGZ-null* MARCM clones. Dl^{+} staining is used to visualize the stem cells (**q**). $Prospero^{+}$ (Pros+) staining is used to mark EEs (**r**). NRE-$LacZ^{+}$ is used to observe EBs (**s**). White arrows indicate ECs. GFP indicates ISCs (green). DAPI stained nuclei (blue). Scale bars represent 5 μm (**c**, **e, j**), 25μm (**m, p, q, r, s**). Bars are mean ± SD. *P* values were calculated by two-tailed, unpaired Student's *t* test. Source data are provided as a Source Data file.

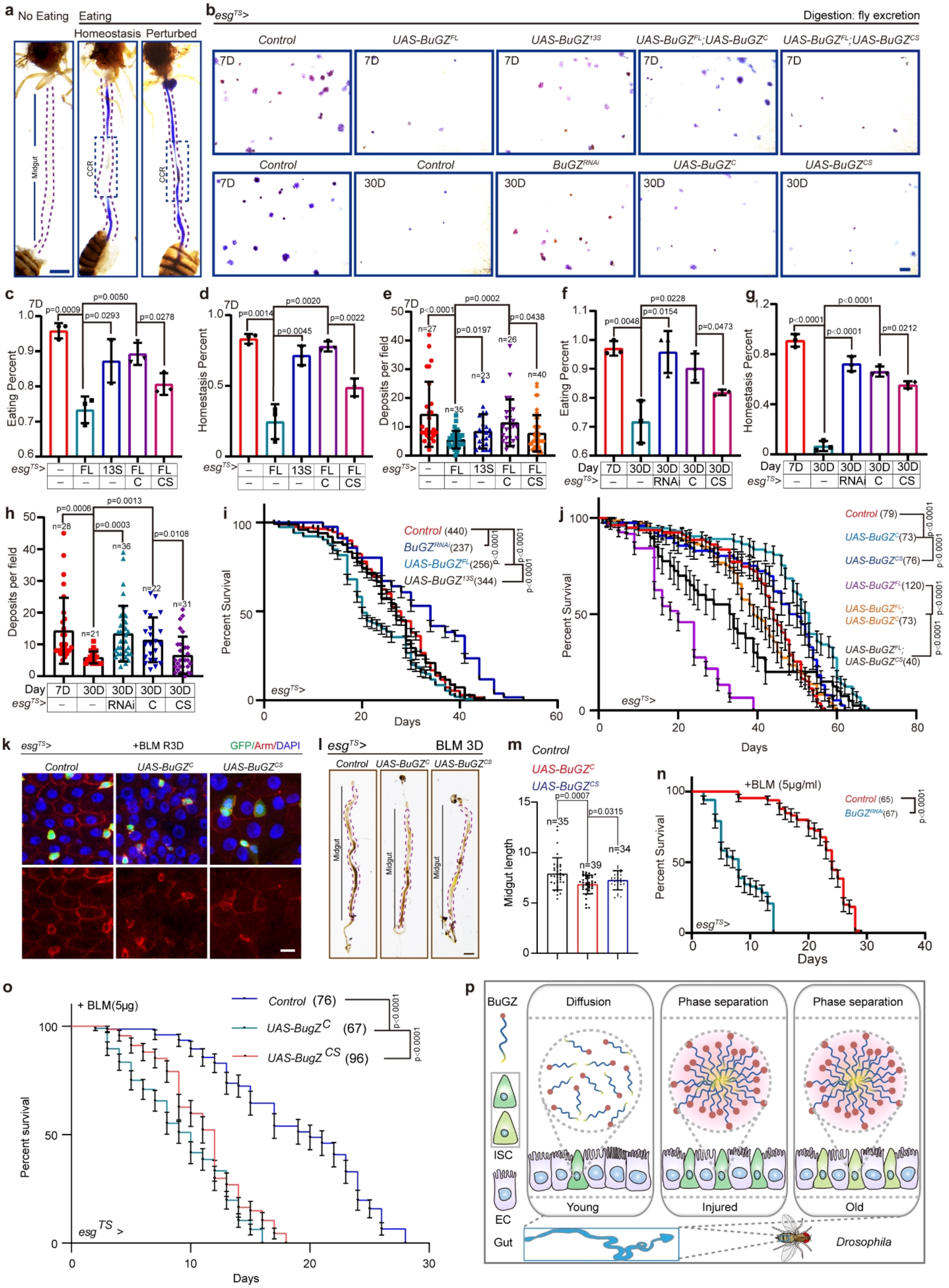

Fig. 4. BuGZ and its phase separation affects the aging of *Drosophila* mediated by gut

digestive functions. a Images of *Drosophila* midguts treated with Bromophenol blue. Treatment flies are divided into food eating or no eating. Moreover, there are two categories among eating: homeostasis, a well-defined acidic copper cell region (CCR) flanked by basic (blue colored) anterior and posterior midgut; and perturbed which whole gut is basic. Scale bars represent 10 μm. **b, c, d, e** LLPS of BuGZ promotes *Drosophila* intestine aging. Excretion of *Drosophila* (**b**). Scale bars represent 1 mm. Ratio of eating flies, intestinal homeostasis categories and excretion of young (7D) flies (**c, d, e** two-tailed unpaired Student's *t*-test). **f, g, h** BuGZ depletion reverses the decline of intestinal digestive functions in aged *Drosophila*. Ratio of eating flies, intestinal homeostasis categories and excretion of young (7D) or old (30D) flies (two-tailed unpaired Student's *t*-test). **i, j** BuGZ or its phase separation regulates *Drosophila* lifespan. Percentage of survival of adults with normal food feeding (statistical significance among genotypes was calculated with a chi-square log-rank test). **k** Midguts were co-stained for GFP (green) and Arm (red). GFP indicates esg^{+} cells. **l, m** Bright field images of *Drosophila* midguts. The dashed red line indicates the position of the midgut (**l**). Midgut length in BLM-treatment *Drosophila* (**m**, *Control*: n=35; $BuGZ^{C}$: n=39; $BuGZ^{CS}$: n=34, two-tailed unpaired Student's *t*-test). **n, o** BuGZ depletion, $BuGZ^{C}$ and $BuGZ^{CS}$ overexpression in ISCs of *Drosophila* with BLM-induced chronic injury leads to higher mortality. Percentage of survival rate after 5μg/ml BLM treatment. (**n**, statistical significance among genotypes was calculated with a chi-square log-rank test). **p** A schematic summary: BuGZ undergoes phase separation in injured- or aged-stem cells to destroy the replenishment of intestinal injury and shorten the lifespan of *Drosophila*. Bars are mean ± SD. Scale bars represent 10 μm (**k**), 0.5mm (**l**). Source data are provided as a Source Data file.

a

Gene name
Nono
Skiv212
Sfpq
Ddx5
Pabp1
Ybx1
Hnrpa2b1;Hnrpa2b1
Rbm14
Hnrnpa1
Hnmpu
Hnrnpa3
Igf2bp3
Sap155
Sf3b3
Sf3a1
Hnrpq
Sfrs1
Sfrs6

b

esg ^{TS}		
**RNAi**	**Phenotype (-BLM)**	**Rescued the phenotype of *BuGZ* RNAi (+BLM)**
Ime4	Promote proliferation of ISCs	Y(***)
Mettl14	Promote proliferation of ISCs	Y(***)
Fl(2)d	No phenotype	N
Nito	No phenotype	N
Vir	No phenotype	N
YT521-B	Promote proliferation of ISCs	Y(***)
CG6422	No phenotype	N
RNAi is driven by *esg* ^{TS}. Y means the phenotype of *BuGZ* RNAi is rescued by indicated gene. N means the phenotype of *BuGZ* RNAi is not rescued by indicated gene. Student's t-test, *p<0.05, **p<0.01, ***p<0.001.		

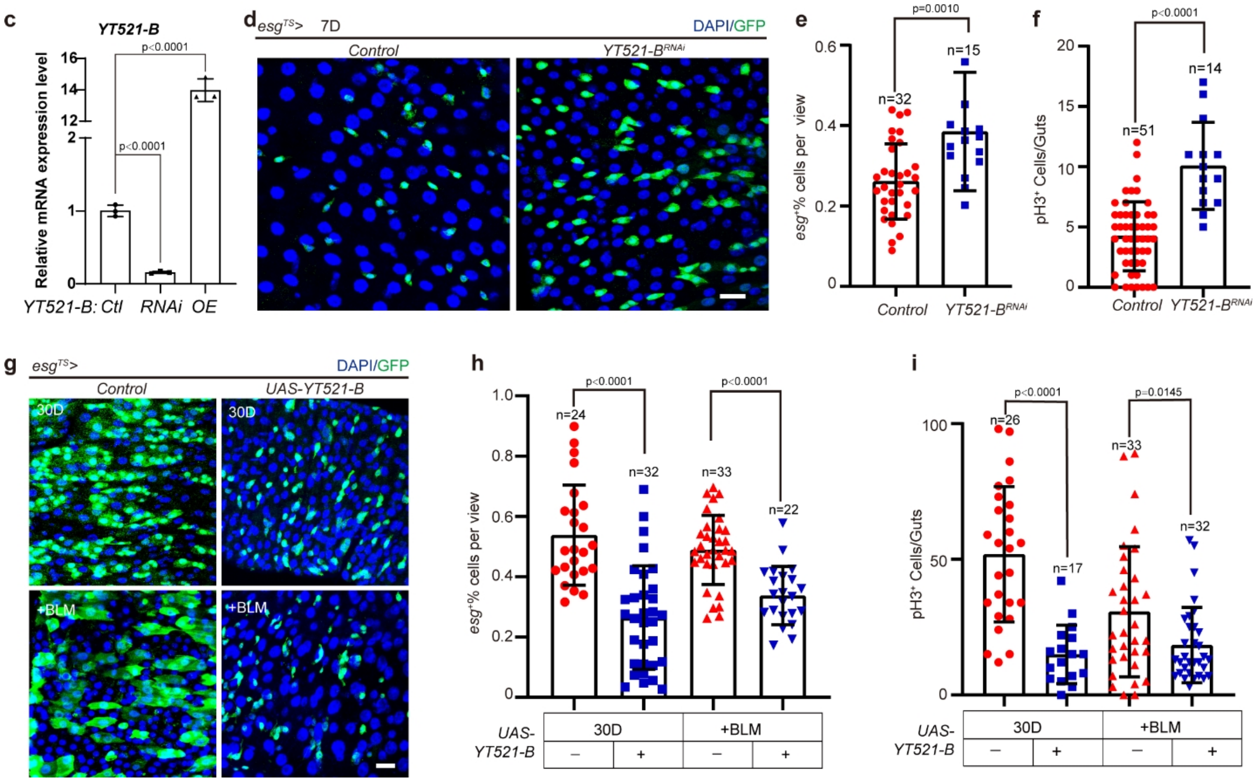

Fig. 5. *YT521-B* RNAi promotes ISC proliferation, wherea YT521-B overexpression inhibits ISC hyperproliferation caused by injury and aging. **a** Previous studies identified BuGZ binding proteins in ESCs with mass spectrometry, and showed strong interactions of BuGZ with

RNA-associated proteins, especially with m^6A-related proteins (Hnrpa2b1, Hnmpa1, Igf2bp3) highlighted with yellow color in the table. **b** Candidate genetic screen with these m^6A-associated genes to uncover the potential genes that could rescue *BuGZ* RNAi phenotype in regulating the ISC proliferation after gut injury, which revealed that only Ime4, Mettl14, and YT521-B depletions reversed the BuGZ depletion phenotype in the screen. **c** YT521-B level in *YT521-B* RNAi and overexpression. **d, e, f** YT*521-B* RNAi promotes ISC proliferation. Representative images of *esg*$^+$ cells (**d**). Ratio of *esg*$^+$ cells to DAPI cells (**e**, *Control* (*UAS-LacZ*): 32 views; *YT521-B*^{RNAi}: 15 views). Number of pH3$^+$ cells per midgut (**f**, *Control* (*UAS-LacZ*): 51 guts; *YT521-B*^{RNAi}: 14 guts). GFP indicates *esg*$^+$ cells (green). **g, h, i** Overexpressing *YT521-B* rescues ISC hyperproliferation caused by aging or gut injury. Immunofluorescence images of *esg*$^+$ cells (**g**). Ratio of *esg*$^+$ cells to DAPI cells (**h**, from left to right are 24, 32, 33, 22 views). Number of pH3$^+$ cells per midgut (**i,** from left to right are 26, 17, 33, 32 guts). GFP indicates *esg*$^+$ cells (green). Scale bars represent 25 μm. View size is 3.4×10^4 μM^2. Bars are mean ± SD. *P* values were calculated by two-tailed, unpaired Student's *t* test. Source data are provided as a Source Data file.

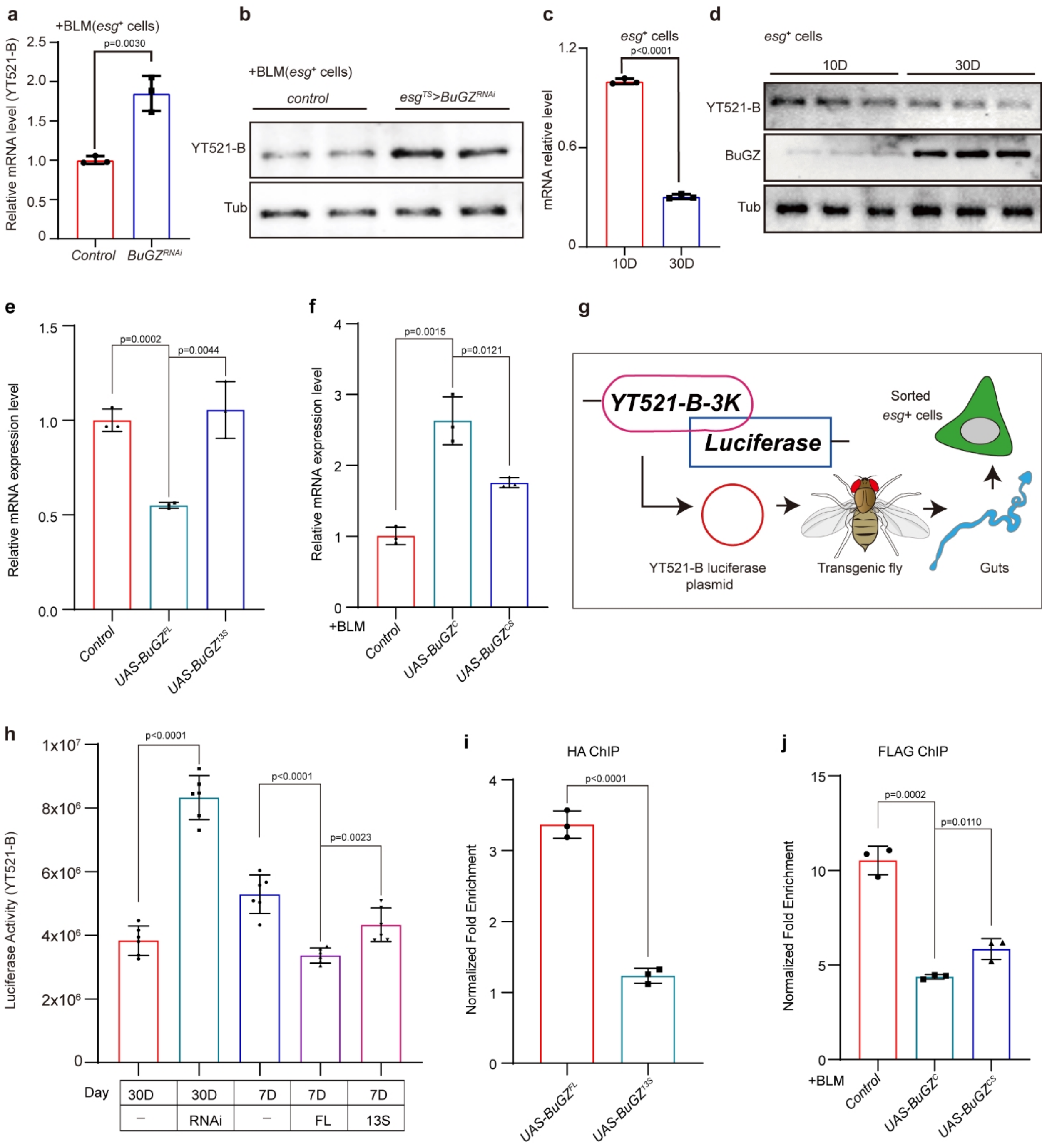

Fig. 6. BuGZ and its condensation property prevent the transcription of m^{6}A reader YT521-B. a RT-qPCR shows BuGZ significantly inhibits YT521-B mRNA levels. RT-qPCR is carried out with control and BuGZ-depleted *esg*$^+$ cells after BLM treatment. **b** Western blotting of the protein levels of YT521-B in control (*UAS-LacZ*), BuGZ depletion sorted *esg*$^+$ cells after BLM-induced injury. **c, d** RT-qPCR and western blotting indicate the reduced level of mRNA and

protein of YT521-B in aged (30D) sorted *esg*$^{+}$ cells. **e, f** YT521-B mRNA level was measured by RT-qPCR when BuGZ$^{FL/13S}$, BuGZ$^{C/CS}$ overexpressed in *esg*$^{+}$ cells. **g** Model of YT521-B-promotor-luciferase transgenic flies and following experiments. **h** The YT521-B promotor luciferase activity is activated by BuGZ depletion in aged (30D) *esg*$^{+}$ cells, while is inhibited by overexpressing *BuGZ^{FL}* instead of *BuGZ^{13S}* in young (7D) *esg*$^{+}$ cells. **i** Chip-qPCR data indicated that BuGZ^{FL} exhibited enhanced binding to the YT521-B promoter in contrast to BuGZ^{13S}. HA antibody was used for Chip assay. **j** BuGZ^{C} overexpression suppressed the binding of the YT521-B promoter to BuGZ in FLAG-BuGZ knock-in flies under BLM treatment, whereas BuGZ^{CS} overexpression displayed diminished effect on attenuating the binding of BuGZ to the YT521-B promotor in relative to BuGZ^{C}. Bars are mean ± SD of 3 biologically independent experiments). *P* values were calculated by two-tailed, unpaired Student's *t* test. Source data are provided as a Source Data file.

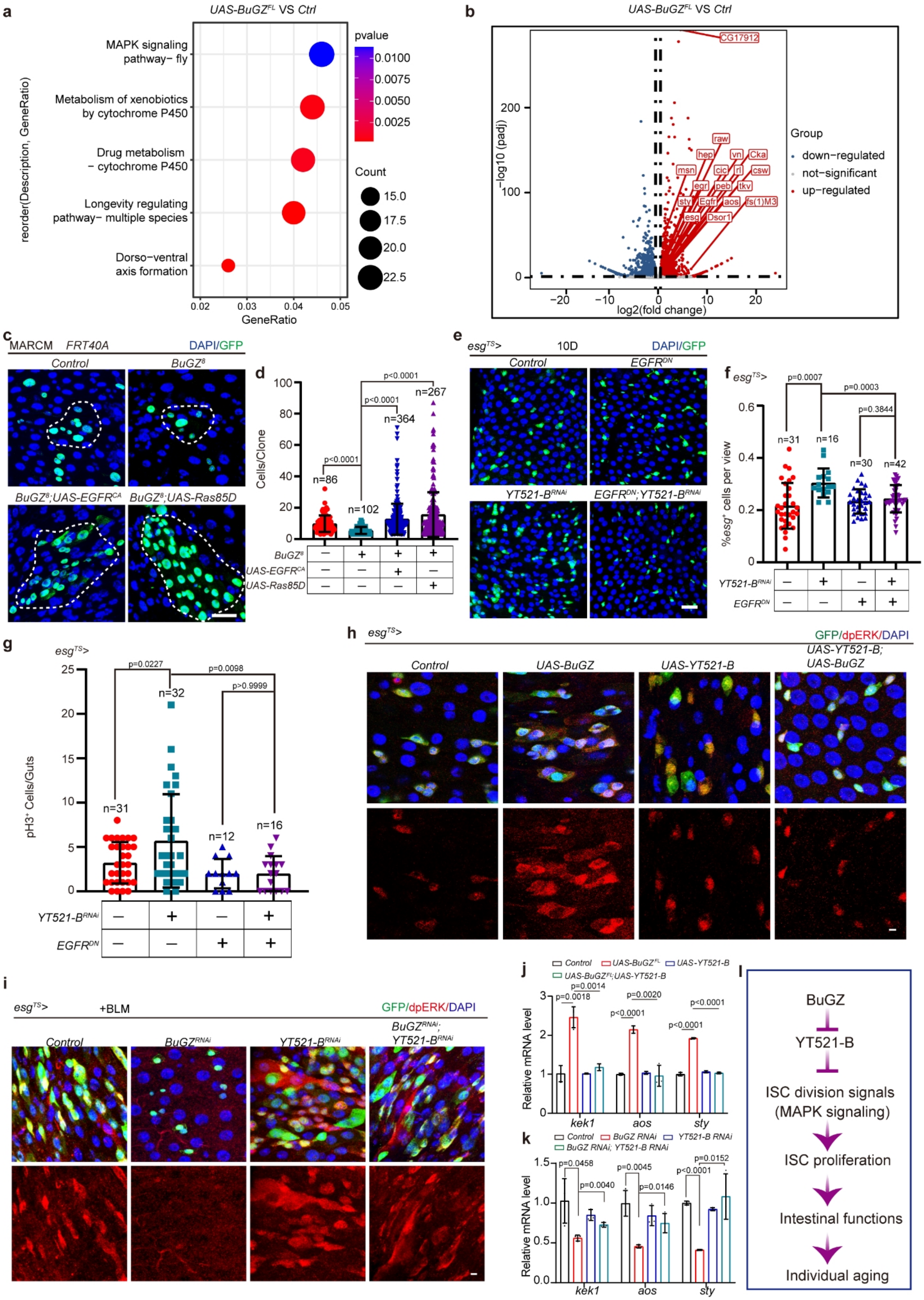
a
UAS-BuGZ^FL VS Ctrl
MAPK signaling pathway− fly
Metabolism of xenobiotics by cytochrome P450
Drug metabolism − cytochrome P450
Longevity regulating pathway− multiple species
Dorso−ventral axis formation
reorder(Description, GeneRatio)
GeneRatio
pvalue
0.0100
0.0075
0.0050
0.0025
Count
15.0
17.5
20.0
22.5
b
UAS-BuGZ^FL VS Ctrl
CG17912
raw
hep
vn
Cka
msn
cic
rl
csw
egr
peb
tkv
sty
Egfr
aos
fs(1)M3
esg
Dsor1
−log10 (padj)
log2(fold change)
Group
down−regulated
not−significant
up−regulated
c
MARCM FRT40A
DAPI/GFP
Control
BuGZ^8
BuGZ^8;UAS-EGFR^CA
BuGZ^8;UAS-Ras85D
d
Cells/Clone
p<0.0001
n=86
n=102
n=364
n=267
BuGZ^8
UAS-EGFR^CA
UAS-Ras85D
e
esg^TS>
10D
DAPI/GFP
Control
EGFR^DN
YT521-B^RNAi
EGFR^DN;YT521-B^RNAi
f
esg^TS>
p=0.0007
p=0.0003
p=0.3844
n=31
n=16
n=30
n=42
%esg+ cells per view
YT521-B^RNAi
EGFR^DN
g
esg^TS>
p=0.0227
p=0.0098
p>0.9999
n=32
n=31
n=12
n=16
pH3+ Cells/Guts
YT521-B^RNAi
EGFR^DN
h
esg^TS>
GFP/dpERK/DAPI
Control
UAS-BuGZ
UAS-YT521-B
UAS-YT521-B; UAS-BuGZ
i
esg^TS>
+BLM
GFP/dpERK/DAPI
Control
BuGZ^RNAi
YT521-B^RNAi
BuGZ^RNAi; YT521-B^RNAi
j
Control
UAS-BuGZ^FL
UAS-YT521-B
UAS-BuGZ^FL;UAS-YT521-B
p=0.0018
p=0.0014
p<0.0001
p=0.0020
p<0.0001
p<0.0001
Relative mRNA level
kek1
aos
sty
k
Control
BuGZ RNAi
YT521-B RNAi
BuGZ RNAi; YT521-B RNAi
p=0.0458
p=0.0040
p=0.0045
p=0.0146
p<0.0001
p=0.0152
Relative mRNA level
kek1
aos
sty
l
BuGZ
YT521-B
ISC division signals (MAPK signaling)
ISC proliferation
Intestinal functions
Individual aging

Fig. 7. BuGZ regulates ISC proliferation via the YT521-B-mediated MAPK signaling pathway. a KEGG pathway enrichment analysis of up-regulated genes in pair-wise comparison of control (*UAS-LacZ*) to *BuGZ*^{FL} overexpression using *esg*^{TS} (q < 0.05). Both adjusted P-value and gene ratio denote the significance of the respective pathway. **b** Volcano plots of differentially expressed genes in pair-wise comparison of control (*UAS-LacZ*) and overexpression of *BuGZ*^{FL}. Blue symbols indicate significantly downregulated mass bins, red symbols indicate significantly upregulated mass bins, and gray symbols indicate mass bins that were not significantly changed. **c, d** The regulators of MAPK signaling pathways can fully rescue the reduced clone cells caused by BuGZ deletion. Images of MARCM clones (green, outlined by white dotted lines) (**c**). Cell number per clone (**d**, *Control*: n=86 clones; *BuGZ*^{8}: n=102 clones; *BuGZ*^{8}*; UAS-Ras85D*: n=364 clones; *BuGZ*^{8}*; UAS-EGFR*^{CA}; n=267 clones). GFP indicates ISCs (green). **e, f, g** The hyperproliferation of ISCs caused by YT521-B depletion can be rescued by the key factors of MAPK signaling pathways. Images of *esg*$^{+}$ cells (**e**). Ratio of *esg*$^{+}$ cells to DAPI (**f**, *Control* (*UAS-LacZ*): n=31 views; *YT521-B*^{RNAi}: n=16 views; *UAS-EGFR*^{DN}: n=30 views; *UAS-EGFR*^{DN}*; YT521-B*^{RNAi}*;* n=42 views). pH3^{+} number per midgut (**g**, from left to right are 31, 32, 12, 16 guts). GFP indicates *esg*$^{+}$ cells (green). **h, i** BuGZ-YT521-B axis regulated the MAPK pathway by modulating the ERK activity. GFP indicates *esg*$^{+}$ cells (green). YT521-B overexpression inhibited the increased dpERK activity caused by BuGZ overexpression (**h**). *YT521-B* RNAi rescued the decrease activity of dpERK result from *BuGZ* RNAi during BLM treatment (**i**). **j, k** BuGZ regulates the mRNA levels of *kek1*, *sty*, and *aos*. (n=3 biologically independent experiments). **l** A schematic diagram of the modulation of BuGZ to ISC proliferation by YT521-B-mediated MAPK signaling pathways. DAPI stained nuclei (blue). Scale bars represent 5 μm (**h, i**), 25 μm (**c, e**). The area of the view size is 3.4×10^{4} μM^{2}. Bars are mean ± SD. *P* values were calculated by two-tailed, unpaired Student's *t* test. Source data are provided as a Source Data file.

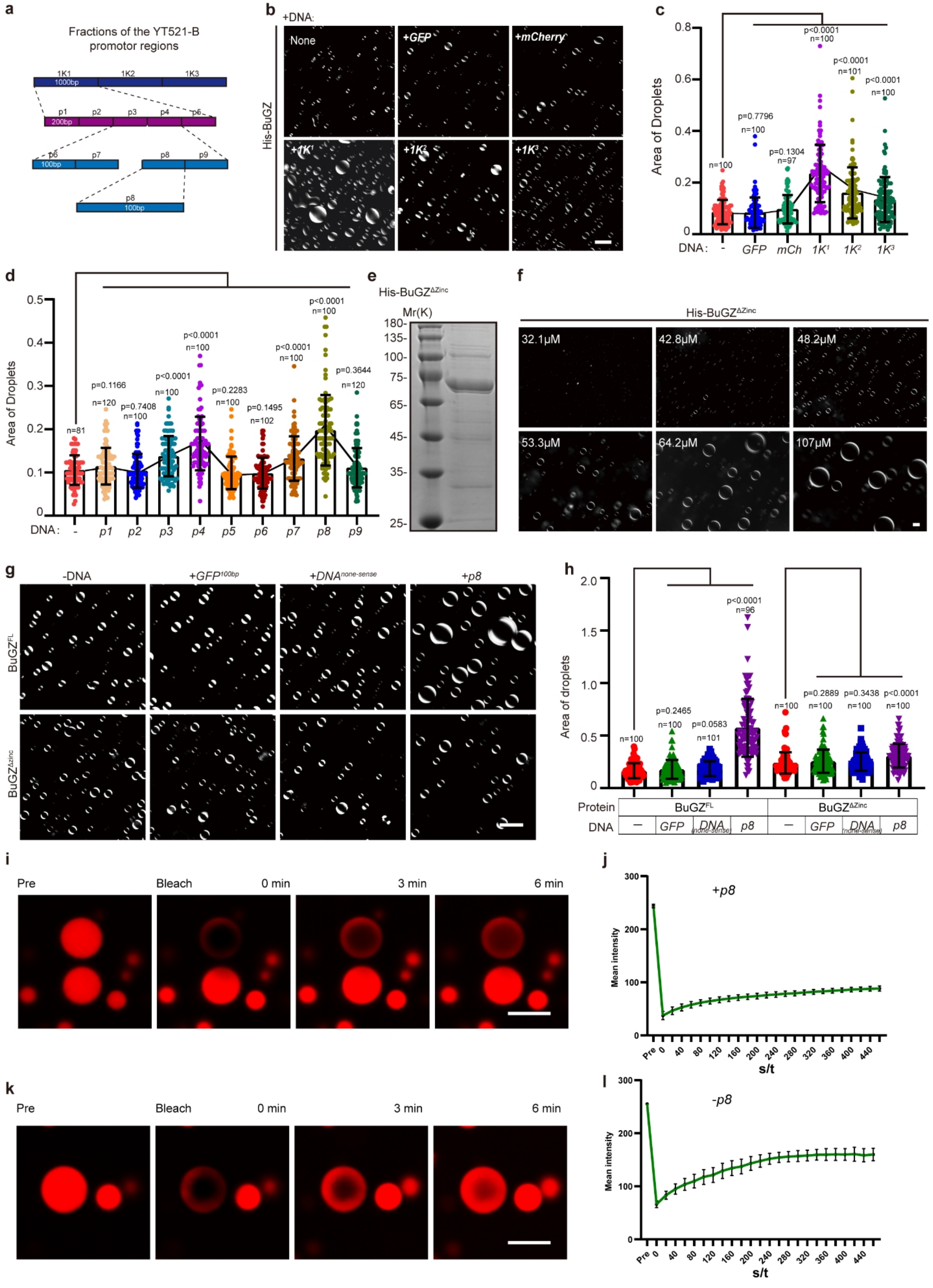
a
Fractions of the YT521-B promotor regions
1K1
1000bp
1K2
1K3
p1
200bp
p2
p3
p4
p5
p6
100bp
p7
p8
p9
p8
100bp
b
+DNA:
None
+GFP
+mCherry
His-BuGZ
+1K1
+1K2
+1K3
c
Area of Droplets
DNA:
-
GFP
mCh
1K1
1K2
1K3
n=100
p=0.7796
n=100
p=0.1304
n=97
p<0.0001
n=100
p<0.0001
n=101
p<0.0001
n=100
d
Area of Droplets
DNA:
-
p1
p2
p3
p4
p5
p6
p7
p8
p9
n=81
p=0.1166
n=120
p=0.7408
n=100
p<0.0001
n=100
p<0.0001
n=100
p=0.2283
n=100
p=0.1495
n=102
p<0.0001
n=100
p<0.0001
n=100
p=0.3644
n=120
e
His-BuGZΔZinc
Mr(K)
180-
135-
100-
75-
65-
45-
35-
25-
f
His-BuGZΔZinc
32.1μM
42.8μM
48.2μM
53.3μM
64.2μM
107μM
g
-DNA
+GFP100bp
+DNAnone-sense
+p8
BuGZFL
BuGZΔZinc
h
Area of droplets
n=100
p=0.2465
n=100
p=0.0583
n=101
p<0.0001
n=96
n=100
p=0.2889
n=100
p=0.3438
n=100
p<0.0001
n=100
Protein
BuGZFL
BuGZΔZinc
DNA
—
GFP
DNA (none-sense)
p8
i
Pre
Bleach
0 min
3 min
6 min
j
+p8
Mean intensity
s/t
k
Pre
Bleach
0 min
3 min
6 min
l
-p8
Mean intensity
s/t

Fig. 8. YT521-B promotor increases BuGZ^{FL} phase separation. a Schematic diagram of screening fragment of YT521-B promotor from 1000 bp (1K) to 100 bp. **b, c, d** Droplets of dBuGZ^{FL} are significantly promoted by *YT521-B* fragments. Area of droplets (**c**, *-DNA*: n=100 droplets; *GFP*: n=100 droplets; *mCherry*: n=97 droplets; *1K*^{1}: n=100 droplets; *1K*^{2}: n=101 droplets; *1K*^{3}: n=100 droplets, His-dBuGZ: 16.7 μM; DNA: 10 μM. **d,** from left to right are: 81, 120, 100, 100, 100, 100, 102, 100, 100, 120 droplets, His-dBuGZ concentration is 8.3 μM and the concentration of DNA is 10 μM). Scale bars represent 10 μm. **e** SDS-PAGE shows purified His-dBuGZ$^{\Delta zinc}$ from *E.coli*. **f** Concentration-dependent phase separation of His-dBuGZ$^{\Delta zinc}$. **g, h** YT521-B promotor fragments promote the size of droplets of BuGZ^{FL} instead of BuGZ$^{\Delta zinc}$. Images of droplets of BuGZ^{FL} (16.7μM) and BuGZ$^{\Delta zinc}$ (16.7μM) with or without 10μM DNA (*GFP*, *DNA*$^{non\text{-}sense}$, *p8*) (**g**). Area of BuGZ ^{FL} and BuGZ$^{\Delta zinc}$ droplets with or without 10μM DNA (*GFP*, *DNA*$^{non\text{-}sense}$, *p8*) (**h**, from left to right are 100, 100, 101, 96, 100, 100, 100, 100 droplets). **i, j, k, l** FARP of scarlet-BuGZ with or without adding *P*^{8} fragment. Scale bars represent 10 μm. Bars are mean ± SD. *P* values were calculated by two-tailed, unpaired Student's *t* test. Source data are provided as a Source Data file.

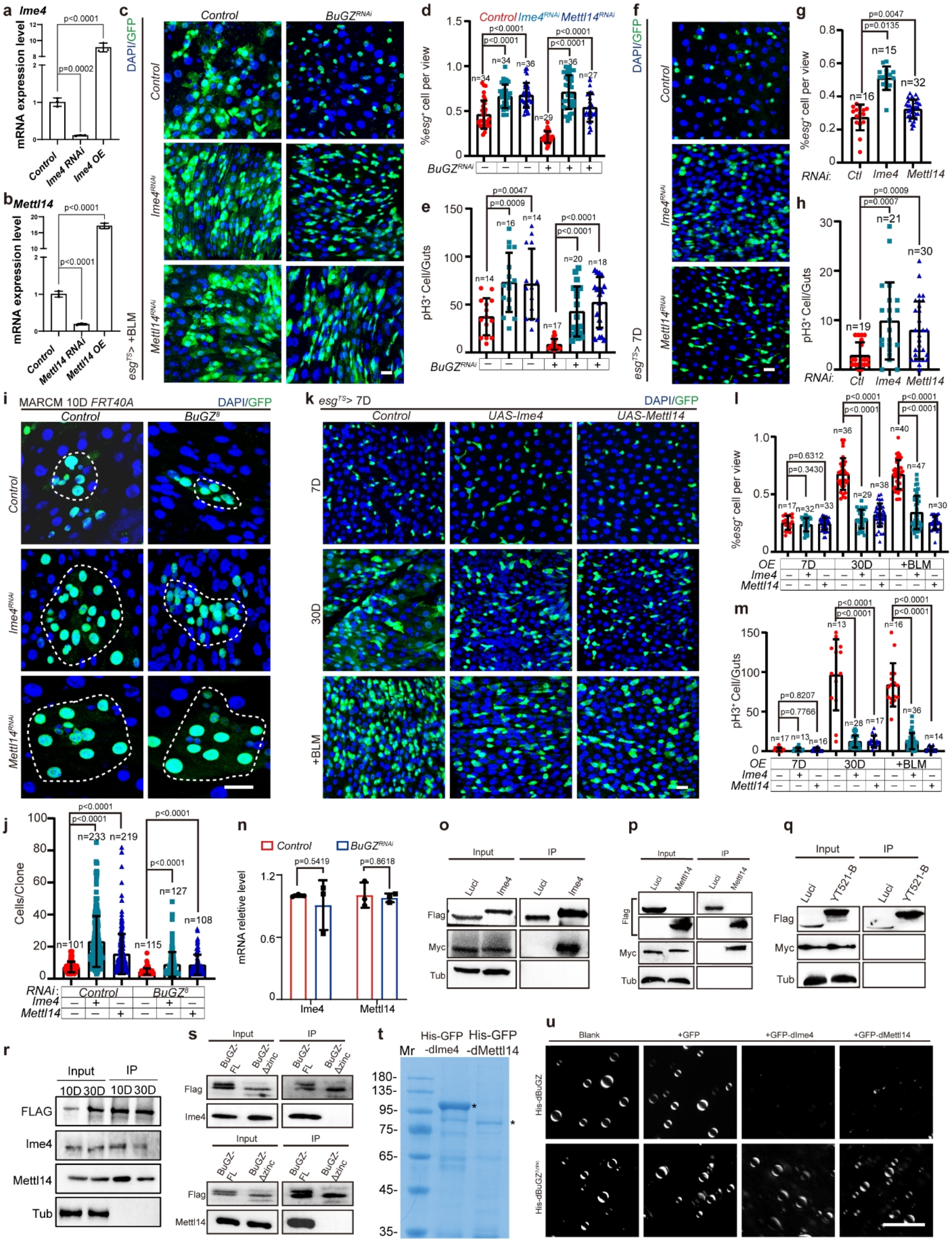

Fig. 9. Ime4/Mettl14 interacts with BuGZ to inhibit its phase separation mediated ISC proliferation. a, b Ime4/Mettl14 mRNA level (n=3 biologically independent experiments). **c, d,**

e Ime4/Mettl14 restores ISC proliferation defect caused by BuGZ depletion and promotes ISCs proliferation after injury. Ratio of *esg*$^+$ cells to DAPI (**d**, from left to right are :34, 34, 36, 29, 36, 27 views). Number of pH3$^+$ cells per midgut (**e**, from left to right are :14, 16, 14, 17, 20, 18 guts). GFP indicates *esg*$^+$ cells (green). **f, g, h** Ime4/Mettl14 depletion promotes ISC proliferation in young *Drosophila*. Ratio of *esg*$^+$ cells to DAPI (**g**, from left to right: 16, 15, 32 views). Number of pH3$^+$ cells per midgut (**h**, from left to right: 19, 21, 30 guts). GFP indicates *esg*$^+$ cells (green). **i, j** Ime4 and Mettl14 inhibition result in bigger clones and rescue *BuGZ* RNAi induced ISC proliferation defect. Number of cells per clone (**j**, from left to right are 101, 233, 219, 115, 127, 108 clones). GFP indicates ISCs (green). **k, l, m** Ime4/Mettl14 overexpressing prevents hyperproliferation of ISCs caused by aging or injury. Ratio of *esg*$^+$ cells to DAPI cells (**l**, from left to right are 17, 32, 33, 36, 29, 38, 40, 47, 30 views). Number of pH3 $^+$ cells per midgut (**m**, from left to right are 17, 13, 16, 13, 28, 17, 16, 36,14 guts). GFP indicates *esg*$^+$ cells (green). **n** RT-qPCR indicates BuGZ does not affect transcription of Ime4 and Mettl14 in injured-*esg*+ cells. (n=3 biologically independent experiments). **o, p, q** BuGZ interacts with Ime4, Mettl14, but not YT521-B. **r** Ime4/Mettl14 interacted with BuGZ^{FL} upon aging. **s** Ime4/Mettl14 not interacts with BuGZ$^{\Delta zinc}$. **t** His-EGFP-dIme4/His-EGFP-dMettl14 protein purified from High five cells. Asterisks indicate protein location. **u** Droplets of dBuGZ-FL/Δzinc are inhibited by His-GFP-dIme4/His-GFP-dMettl14. dBuGZ, His-GFP, His-GFP-dIme4, His-GFP-Mettl14: 15 μM. Scale bars represent 25 μm (**c, f, i, k**), 10 μm (**u**). View size is 3.4×10^4 μM^2. Bars are mean ± SD. *P* values were calculated by two-tailed, unpaired Student's *t* test. Source data are provided as a Source Data file.

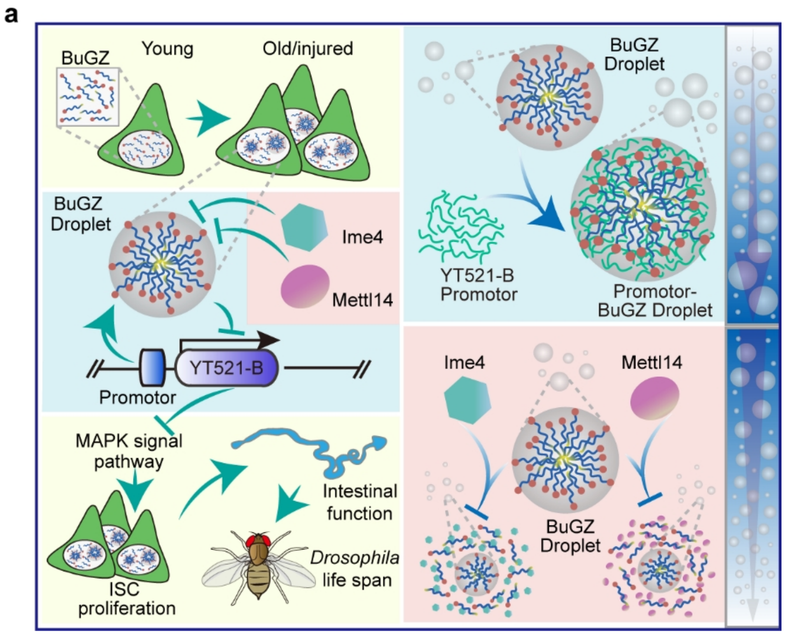

Fig. 10. Phase separation controls intestinal stem cell proliferation upon gut regeneration and aging.

a, BuGZ shows age- and injury-associated condensation in *Drosophila* ISC nuclei. BuGZ condensation promotes ISC proliferation by control YT521-B transcription mediated MAPK signal pathway to affect *Drosophila* gut repair and longevity. Meanwhile, Ime4 and Mettl14 interact with BuGZ to inhibit BuGZ phase separation in *Drosophila* ISCs, attenuate the transcription inhibitory property of BuGZ, promote the transcription of m^6A reader YT521-B, the downstream of BuGZ, and prevent the proliferation of ISCs through the MAPK pathway.
