# Supplementary material for: Phase separation of BuGZ regulates gut regeneration and aging through interaction with m6A regulators
Source: Nat Commun. 2023 Oct 23;14:6700. doi: 10.1038/s41467-023-42474-1 (PMC10593810; doi:10.1038/s41467-023-42474-1)
Supplement: Supplementary file 3 — Description of Additional Supplementary Files [file 41467_2023_42474_MOESM3_ESM.pdf]

File name: Supplementary Data 1

Description: Primers for RT-qPCR, Constructs, YT521-B fragments and probe for in situ hybridization.

File name: Supplementary Data 2

Description: List of changed genes in BuGZFL overexpression compared to Control.The differentially expressed genes were determined by using DESeq2 with default parameters. The statistical test used for data analysis by DESeq2 was Wald test. The “padj” in the supplementary table 2 was Benjamini-Hochberg adjusted p-values.

File name: Supplementary Data 3

Description: Full Drosophila genotypes as they appear in each figure panel.
