# Supplementary material for: Phase separation of BuGZ regulates gut regeneration and aging through interaction with m6A regulators
Source: Nat Commun. 2023 Oct 23;14:6700. doi: 10.1038/s41467-023-42474-1 (PMC10593810; doi:10.1038/s41467-023-42474-1)
Supplement: Supplementary file 7 — Reporting Summary [file 41467_2023_42474_MOESM7_ESM.pdf]

nature portfolio

Corresponding author(s): Hao Jiang and Haiyang Chen

Last updated by author(s): Sep 27, 2023

Reporting Summary

Nature Portfolio wishes to improve the reproducibility of the work that we publish. This form provides structure for consistency and transparency in reporting. For further information on Nature Portfolio policies, see our Editorial Policies and the Editorial Policy Checklist.

Statistics

For all statistical analyses, confirm that the following items are present in the figure legend, table legend, main text, or Methods section.

n/a	Confirmed	
☐	☒	The exact sample size (*n*) for each experimental group/condition, given as a discrete number and unit of measurement
☐	☒	A statement on whether measurements were taken from distinct samples or whether the same sample was measured repeatedly
☐	☒	The statistical test(s) used AND whether they are one- or two-sided<br>*Only common tests should be described solely by name; describe more complex techniques in the Methods section.*
☒	☐	A description of all covariates tested
☒	☐	A description of any assumptions or corrections, such as tests of normality and adjustment for multiple comparisons
☐	☒	A full description of the statistical parameters including central tendency (e.g. means) or other basic estimates (e.g. regression coefficient) AND variation (e.g. standard deviation) or associated estimates of uncertainty (e.g. confidence intervals)
☐	☒	For null hypothesis testing, the test statistic (e.g. *F*, *t*, *r*) with confidence intervals, effect sizes, degrees of freedom and *P* value noted<br>*Give P values as exact values whenever suitable.*
☒	☐	For Bayesian analysis, information on the choice of priors and Markov chain Monte Carlo settings
☒	☐	For hierarchical and complex designs, identification of the appropriate level for tests and full reporting of outcomes
☒	☐	Estimates of effect sizes (e.g. Cohen's *d*, Pearson's *r*), indicating how they were calculated

Our web collection on statistics for biologists contains articles on many of the points above.

Software and code

Policy information about availability of computer code

Data collection	TCS-SP8 confocal microscope (Leica TCS-SP8, v3.5.5.19976) was used to imaging collection; Upright Microscopes (Leica DM6 B) was used to collect droplets images; Fluorescence stereo microscopes (Leica M205 FA, v3.7.3.23245) was used to collect bright fieldimages; C1000 Touch Thermal Cycler (v4.1.2433.12.19) was used to RT-qPCR; Multifunctional microplate reader (CLARIOstar,v5.40 R3) was used for luciferise data collection; the western blots were detected using Bio-rad ChemiDoc XRS+ (Image Lab, v6.0.1).
Data analysis	The initial quality control of the raw fastq files was performed using FastQC (v0.11.9, http://www.bioinformatics.babraham.ac.uk/projects/fastqc/), and the adapter sequences were removed by Trim Galore (v0.6.7, https://www.bioinformatics.babraham.ac.uk/projects/tri-m_galore/). The trimmed RNA-seq reads were aligned to the Drosophila reference sequence Ensembl build BDGP6 (https://support.illumina.com/sequencing/sequencing_software/igeno-me.html) with hisat2 (v2.20,45). Aligned reads were then sorted by chromosome position using SAMtools (v1.15.1, https://sourceforge.net/projects/samtools/files/samtools/) and converted into bam files. Gene raw counts matrix per sample was computed using StringTie (v2.2.1, https://ccb.jhu.edu/software/stringtie/). Gene symbols were annotated by Drosophila melanogaster BDGP6 from Ensembl (https://support.illumina.com/sequencing/sequencing_soft-ware/igenome.html). Differentially expressed genes were determined using DESeq2 (v1.26.0,46) with default parameters. Genes were considered differentially expressed if the absolute value of the log2 fold change was greater than 0.5 between modification (i.e. Overexpressed) and control at padj < 0.05. Downstream pathway analysis was performed via clusterProfiler (v3.14.3,47) using genes at padj < 0.05. Fluorescence intensity of region of interest (ROI) or cell was calculated using software LAS X (3.4.2.18368).<br>GraphPad Prism 8 (v8.0.2) were used as statistical softwares. A p-value of 0.05 or less was considered to be statistically significant. Results of lifespan experiments were analyzed using Kaplan-Meier survival analysis and compared among groups scoring for significance using long-rank test.

For manuscripts utilizing custom algorithms or software that are central to the research but not yet described in published literature, software must be made available to editors and reviewers. We strongly encourage code deposition in a community repository (e.g. GitHub). See the Nature Portfolio guidelines for submitting code & software for further information.

Data

Policy information about availability of data

All manuscripts must include a data availability statement. This statement should provide the following information, where applicable:

- Accession codes, unique identifiers, or web links for publicly available datasets
- A description of any restrictions on data availability
- For clinical datasets or third party data, please ensure that the statement adheres to our policy

The RNA-seq data generated in this study have been deposited in Sequence ReadArchive repository database under accession code PRJNA885069 [https://www.ncbi.nlm.nih.gov/bioproject/PRJNA885069]. The up and down-regulated genes are generated in this study are provided in Supplementary datasheet (Table S2). Source data are provided with this paper.

Research involving human participants, their data, or biological material

Policy information about studies with human participants or human data. See also policy information about sex, gender (identity/presentation), and sexual orientation and race, ethnicity and racism.

Reporting on sex and gender	N/A
Reporting on race, ethnicity, or other socially relevant groupings	N/A
Population characteristics	N/A
Recruitment	N/A
Ethics oversight	N/A

Note that full information on the approval of the study protocol must also be provided in the manuscript.

Field-specific reporting

Please select the one below that is the best fit for your research. If you are not sure, read the appropriate sections before making your selection.

☒ Life sciences ☐ Behavioural & social sciences ☐ Ecological, evolutionary & environmental sciences

For a reference copy of the document with all sections, see nature.com/documents/nr-reporting-summary-flat.pdf

Life sciences study design

All studies must disclose on these points even when the disclosure is negative.

Sample size	Sample sizes were determined based on variations between different genotypes and treatments. At least 2-3 replicates were carried out in each experiment.
Data exclusions	No data were excluded from the analysis.
Replication	All experiment are replicated 2-3 times and representative results are shown in the manuscript.
Randomization	The same genotype flies were collected and random grouped into batches.
Blinding	Blinding was performed in all quantification. other experiments were performed non blind as the different genotypes of fly should be staining with appropriate antibodies.

Reporting for specific materials, systems and methods

We require information from authors about some types of materials, experimental systems and methods used in many studies. Here, indicate whether each material, system or method listed is relevant to your study. If you are not sure if a list item applies to your research, read the appropriate section before selecting a response.

Materials & experimental systems

n/a	Involved in the study
	☒ Antibodies
	☒ Eukaryotic cell lines
☒	☐ Palaeontology and archaeology
	☒ Animals and other organisms
☒	☐ Clinical data
☒	☐ Dual use research of concern
☒	☐ Plants

Methods

n/a	Involved in the study
☒	☐ ChIP-seq
☒	☐ Flow cytometry
☒	☐ MRI-based neuroimaging

Antibodies

Antibodies used

Primary antibodies used in this study were listed as follows:
Chicken polyclonal anti-GFP (Abcam; Cat# ab13970; RRID: AB_300798; 1:1000); Rabbit Polyclonal anti-GFP (Proteintech; Cat# 50430-2-AP; RRID: AB_11042881; 1:1000); Rabbit Polyclonal anti-FLAG (Cell Signaling Technology; Cat# 14793; RRID: AB_2572291; 1:1000); Mouse anti-HA (ABclonal; Cat# AE008; RRID: AB_2770404; 1:1000); Mouse anti-Delta (DSHB; Cat# C594.9B; RRID: AB_528194; 1:50); Mouse anti-Prospero (DSHB; Cat# MR1A; RRID: AB_528440; 1:200); Chicken anti-β-Galactosidase (Abcam; Cat# ab9361; RRID: AB_307210; 1:1000); Rabbit polyclonal anti-Fibrillarin (ABclonal; Cat# A13490; RRID: AB_2760353; 1:1000); Rabbit polyclonal anti-SUMO1 (ABclonal; Cat# A2130; RRID: AB_2764149; 1:1000); anti-γH2AvD (Rockland Cat# 600- 401- 914, RRID:AB_828383); anti-TriMethyl-Histone H3-K27 Rabbit pAb (ABclone cat# A2363, RRID: AB_2756439); anti-Hp1α(abcam, cat# ab77256, RRID:AB_1523784)Rabbit monoclonal α-Tubulin (11H10) (Cell Signaling Technology; Cat# 2125; RRID: AB_2619646; 1:1000); Rabbit anti-phosphoHistone H3 (Ser10) (Millipore; Cat# 06-570; RRID: AB_310177; 1:1000); Mouse monoclonal anti-FLAG® M2 antibody (Sigma-Aldrich; Cat# F1804; RRID: AB_262044; 1:1000); Mouse monoclonal anti-Myc (9B11) (Cell Signaling Technology; Cat# 2276; RRID: AB_331783;1:1000); Rabbit anti-HA (C29F4) (Cell Signaling Technology; Cat# 3724, RRID:AB_1549585); Mouse Anti-Drosophila Armadillo Protein Monoclonal Antibody (DHSB; Cat# N2 7A1; RRID:AB_528089; 1:100).

Other antibodies used in this paper are as follows:
HRP-mouse anti-rabbit (Jackson ImmunoResearch Labs; Cat# 211-032-171; 1:10000); HRP-Goat anti-mouse (Jackson ImmunoResearch Labs; Cat# 115-035-174; 1:10000); Goat anti-Mouse IgG (H+L) Cross-Adsorbed Secondary Antibody, Alexa Fluor™ 488 , Goat anti-Mouse IgG (H+L) Cross-Adsorbed Secondary Antibody, Alexa Fluor™ 568, Goat anti-Mouse IgG (H+L) Cross-Adsorbed Secondary Antibody, Alexa Fluor™ 647, Goat anti-Rabbit IgG (H+L) Highly Cross-Adsorbed Secondary Antibody, Alexa Fluor™ 488, Goat anti-Rabbit IgG (H+L) Cross-Adsorbed Secondary Antibody, Alexa Fluor™ 568, Goat anti-Rabbit IgG (H+L) Highly Cross-Adsorbed Secondary Antibody, Alexa Fluor™ 647, Goat anti-Chicken IgY (H+L) Secondary Antibody, Alexa Fluor 488 (Thermo Fisher Scientific; Cat# A-11001, Cat# A-11004, Cat# A-21235, Cat# A-11034, Cat# A-11011, Cat# A-21245, Cat# A-11039; 1:2000).

Validation

Most of antibodies used in this study have been validated by previous work specifically in Drosophila midgut.
Chicken polyclonal anti-GFP (Abcam; Cat# ab13970)/Mouse anti-Delta (DSHB; Cat# C594.9B)/Mouse anti-Prospero (DSHB; Cat# MR1A)/ Chicken anti-β-Galactosidase (Abcam; Cat# ab9361)/Rabbit monoclonal α-Tubulin (11H10) (Cell Signaling Technology; Cat# 2125)/Rabbit anti-phosphoHistone H3 (Ser10) (Millipore; Cat# 06-570)/Mouse anti-Armadillo antibody are validated in "Du, G. et al. Peroxisome Elevation Induces Stem Cell Differentiation and Intestinal Epithelial Repair. Dev Cell 53, 169-184 e111 (2020)".

Rabbit Polyclonal anti-FLAG (Cell Signaling Technology; Cat# 14793) validation stated on supplier' website https://www.cellsignal.cn/products/primary-antibodies/dykddddk-tag-d6w5b-rabbit-mab-binds-to-same-epitope-as-sigma-aldrich-anti-flag-m2-antibody/14793?site-search-type=Products&N=4294956287&Ntt=flag&fromPage=plp;
Rabbit anti-HA (C29F4) (Cell Signaling Technology Cat# 3724) validation stated on supplier' website https://www.cellsignal.com/product/productDetail.jsp?productId=3724;
Mouse anti-HA (ABclonal; Cat# AE008) validation stated on supplier' website https://abclonal.com.cn/catalog/AE008;
Rabbit polyclonal anti-Fibrillarin (ABclonal; Cat# A13490) validation stated on supplier' website https://abclonal.com.cn/catalog/A13490;
Rabbit polyclonal anti-SUMO1 (ABclonal; Cat# A2130) validation stated on supplier' website https://abclonal.com.cn/catalog/A21643;
Rabbit monoclonal α-Tubulin (11H10) (Cell Signaling Technology; Cat# 2125) validation stated on supplier' website https://www.cellsignal.cn/products/primary-antibodies/a-tubulin-11h10-rabbit-mab/2125?site-search type=Products&N=4294956287&Ntt=2125&fromPage=plp&_requestid=4273633/;
Mouse monoclonal anti-FLAG® M2 antibody (Sigma-Aldrich; Cat# F1804) validation stated on supplier' website https://www.sigmaaldrich.cn/CN/zh/product/sigma/f1804;
Mouse monoclonal anti-Myc (9B11) (Cell Signaling Technology; Cat# 2276) is validated by manufacturer as https://www.cellsignal.cn/products/primary-antibodies/myc-tag-9b11-mouse-mab/2276?site-search-type=Products&N=4294956287&Ntt=2276&fromPage=plp.
Rabbit Polyclonal anti-dBuGZ antibody and Rabbit Polyclonal anti-dYT521-B is validated in our manuscript data.

HRP-mouse anti-rabbit (Jackson ImmunoResearch Labs; Cat# 211-032-171; 1:10000) is validated by manufacturer (https://www.jacksonimmuno.com/catalog/products/211-032-171; HRP-Goat anti-mouse (Jackson ImmunoResearch Labs; Cat# 115-035-174; 1:10000);
HRP-goat anti-mouse (Jackson ImmunoResearch Labs; Cat# 115-035-174; 1:10000) is validated by manufacturer (https://www.jacksonimmuno.com/catalog/products/115-035-174);

Goat anti-Mouse IgG (H+L) Cross-Adsorbed Secondary Antibody, Alexa Fluor™ 488 (Thermo Fisher Scientific; Cat# A-11001) is validated by manufacturer (https://www.thermofisher.cn/cn/zh/antibody/product/Goat-anti-Mouse-IgG-H-L-Cross-Adsorbed-Secondary-Antibody-Polyclonal/A-11001);

Goat anti-Mouse IgG (H+L) Cross-Adsorbed Secondary Antibody, Alexa Fluor™ 568 (Thermo Fisher Scientific; Cat# A-11004) is validated by manufacturer (https://www.thermofisher.cn/cn/zh/antibody/product/Goat-anti-Mouse-IgG-H-L-Cross-Adsorbed-Secondary-Antibody-Polyclonal/A-11004);
Goat anti-Mouse IgG (H+L) Cross-Adsorbed Secondary Antibody, Alexa Fluor™ 647 (Thermo Fisher Scientific; Cat# A-21235) is validated by manufacturer (https://www.thermofisher.cn/cn/zh/antibody/product/Goat-anti-Mouse-IgG-H-L-Cross-Adsorbed-Secondary-Antibody-Polyclonal/A-21235);
Goat anti-Rabbit IgG (H+L) Highly Cross-Adsorbed Secondary Antibody, Alexa Fluor™ 488 (Thermo Fisher Scientific; Cat# A-11034) is validated by manufacturer (https://www.thermofisher.cn/cn/zh/antibody/product/Goat-anti-Rabbit-IgG-H-L-Highly-Cross-Adsorbed-Secondary-Antibody-Polyclonal/A-11034);
Goat anti-Rabbit IgG (H+L) Cross-Adsorbed Secondary Antibody, Alexa Fluor™ 568 (Thermo Fisher Scientific; Cat# AA-11011) is validated by manufacturer (https://www.thermofisher.cn/cn/zh/antibody/product/Goat-anti-Rabbit-IgG-H-L-Cross-Adsorbed-Secondary-Antibody-Polyclonal/A-11011);
Goat anti-Rabbit IgG (H+L) Highly Cross-Adsorbed Secondary Antibody, Alexa Fluor™ 647 (Thermo Fisher Scientific; Cat# A-21245) is validated by manufacturer (https://www.thermofisher.cn/cn/zh/antibody/product/Goat-anti-Rabbit-IgG-H-L-Highly-Cross-Adsorbed-Secondary-Antibody-Polyclonal/A-21245);
Goat anti-Chicken IgY (H+L) Secondary Antibody, Alexa Fluor 488 (Thermo Fisher Scientific; Cat# A-11039) are validated by manufacturer (https://www.thermofisher.cn/cn/zh/antibody/product/Goat-anti-Chicken-IgY-H-L-Secondary-Antibody-Polyclonal/A-11039).

Eukaryotic cell lines

Policy information about cell lines and Sex and Gender in Research

Cell line source(s): HEK293 cell (ATCC, #CRL-3216); SF9 cells (ATCC, , #CRL-1711); High FiveTM (Hi5, from Sinobiological company) cells

Authentication: Authentication of the cell line was confirmed by genomic analysis.

Mycoplasma contamination: Cell used in this study are mycoplasma contamination free as confirmed routinely with Mycoplasma Stain Assay Kit (Beyotime)

Commonly misidentified lines (See ICLAC register): No misidentified lines were used in the study.

Animals and other research organisms

Policy information about studies involving animals; ARRIVE guidelines recommended for reporting animal research, and Sex and Gender in Research

Laboratory animals: This study made use of a variety of stocks of Drosophila melanogaster. Stock origin are as follows:
w1118; PBac{BuGZ-GFP.FPTB}VK00033 (BDSC:67713), 386Y-Gal4 line: w[*]; P{w [+mW.hs] =GawB}386Y (BDSC:25410), MARCM line: w[1118]; P{ry[+t7.2]=neoFRT}40A/CyO;P{ry[+t7.2]=ey-FLP.N}6, ry[506] (BDSC:8212), BuGZ RNAi: y1 v1; P{TRiP.JF02830}attP2 (BDSC:27996), UAS-Ime4 line: y1 w*; P{UAS-Mettl3.HA.H}attP40/CyO (BDSC:77882), UAS-YT521-B line: y1 w*; P{UAS-Ythdc1.HA}attP40 (BDSC:77884), UAS-EGFR-DN line: y1 w*; P{UAS-Egfr.DN.B}29-77-1; P{UAS-Egfr.DN.B}29-8-1 (BDSC:5364), UAS-EGFR-CA line: w*; P{Egfr.2.A887T.UAS}8-2 (BDSC:9533), UAS-LacZ line: w[*]; P{w[+mC]=UAS-lacZ.Exel}2 (BDSC:8529), UAS-mCherry line: w[*]; P{w[+mC]=UAS-mCherry.NLS}2; MKRS/TM6B, Tb[1] (BDSC:38425) are obtained from the Bloomington Drosophila Stock Center (BDSC)]:

BuGZ RNAi: P{KK109387}VIE-260B (VDRC: v104498), Ime4 RNAi: w1118; P{GD9882}v20969/TM3 (VDRC: v20969), Mettl14 RNAi: w1118; P{GD16300}v48560 (VDRC: v48560) were obtained from Vienna Drosophila Resource Center (VDRC).

YT521-B RNAi: THU1532; Fl(2)d RNAi: TH02871.N; nito RNAi: THU0697; Vir RNAi: TH02977.N; CG6422 RNAi: TH03062.N. MARCM line: hsFLP, tub-GAL4, UAS-GFP-NLS/FM7; tub-Gal80, FRT40A/CyO (TB00132) were obtained from Tsinghua Fly Center.

esg-GFP (Carnegie Protein Trap line esgCB02017), Flipout line: yw, hsFLP; Act.FRT-CD2-FRT.GAL4, UAS-GFP S65T/CyO, UAS-Ras85D line: w[1118]; ;UAS-Ras85D.V12 were obtained from Allan Spradling. esg-lacZ (esgM5-4) was obtained from Mark Van Doren. NRE-lacZ, esgts-GAL4 line: esg-GAL4, UAS-GFP, tub-Gal80ts/CyO, ISCts-GAL4 line: NRE-lacZ; esg-GAL4, UAS-GFP/CyO; tub-Gal80ts, NRE-Gal80, NREts-GAL4 line: NRE-GAL4, UAS-GFP, tub-Gal80ts/CyO were obtained from Benjamin Ohlstein.

Crosses involving conditional Gal80ts-dependent expression of transgenes including RNAi or gene overexpression were maintained at 18°C until adult offspring were enclosed. Adults were kept at 18°C for 1-3 days before they were shifted to 29°C to turn on the GAL4 system for another 7-10 days or 30 days before dissection. For the experiment, only mated females were used on the Drosophila midguts.

For clone analysis, flies were maintained at 29°C for 24 hours within 2 ~3 days ACI after heat shock, and then these flies were dissected and observed at 10 days ACI.

Wild animals: This study did not involve wild animals.

Reporting on sex: For the experiment, due to the phenotype of male and female is consistent, only mated females were used on the Drosophila midguts.

Field-collected samples: No field collected animal were used in this study

Ethics oversight	No ethics approval or oversight is required for drosophila studies

Note that full information on the approval of the study protocol must also be provided in the manuscript.
